# A cross-sectional survey on the health status and the health-related quality of life of the elderly after flood disaster in Bazhong city, Sichuan, China

**DOI:** 10.1186/s12889-015-1402-5

**Published:** 2015-02-19

**Authors:** Jun Wu, Jian Xiao, Tong Li, Xiaoshan Li, Huamin Sun, Eric PF Chow, Yihua Lu, Tian Tian, Xiaoyan Li, Qi Wang, Xun Zhuang, Lei Zhang

**Affiliations:** Nantong Tumor Hospital Affiliated of Nantong University, Jiangsu, China; Nantong Shipping College, Jiangsu, China; Department of Epidemiology and Biostatistics, School of Public Health, Nantong University, 9 Seyuan Road, Nantong City, 226019 Jiangsu China; The Kirby Institute, the University of New South Wales, Sydney, Australia; Melbourne Sexual Health Centre, Alfred Health, Melbourne, Melbourne, Victoria Australia; Central Clinical School, Faculty of Medicine, Nursing and Health Sciences, Monash University, Melbourne, Victoria Australia; School of Public Health, Wuhan University, Wuhan, China; School of Public Health, Southeast University, No. 82 Chengxian Street, Xuanwu District, Nanjing City 210018, Jiangsu, China

**Keywords:** Flood disaster, Elderly, Health status, Health-related quality of life (HRQoL)

## Abstract

**Background:**

Flood is common in China and causes extensive loss of property and human lives. Elderly is a vulnerable population prone to the detrimental impacts of floods. This survey aims to investigate the health status and the HRQoL of the elderly in Bazhong city after a major flood in 2011.

**Methods:**

A total of 1183 elderly (aged > 60) were surveyed through random sampling from eight villages in Bazhong city. Two-week healthcare-seeking rate and chronic diseases prevalence were recorded anonymously. Health-related quality of life (HRQoL) was measured by the Medical Outcomes Study Short Form-36 (MOS SF-36). Multivariate regression analysis was conducted to determine the associated factors of poor HRQoL.

**Results:**

The two-week healthcare-seeking rate among post-flood Bazhong elderly was significantly higher than the references rate among rural elderly in Sichuan province (59.3% versus 55.7%, χ2 = 5.134, p = 0.013), but Bazhong elderly demonstrated a significantly lower prevalence of chronic disease (33.2% versus 44.4%, χ2 = 48.847, p < 0.001). All dimension scores among Bazhong elderly were significantly lower than the references scores in rural Sichuan elderly. The determinants of poor physical health included older age, singlehood, poor sleep patterns, and chronic diseases and so on.

**Conclusions:**

A marked decline in health status among elderly in Bazhong after the 2011 flood. Post-flood management targeting elderly need to be sensitive to their age, gender, married status and status of chronic diseases.

## Background

China is one of the developing countries most affected by natural disasters in the world. Since 1949, more than 200 million Chinese have suffered from various natural disasters every year. The annual death toll due to natural disasters is about 16,000, and direct economic loss accounts for 3-6% of China’s Gross Domestic Product (GDP) [1]. Major natural disasters not only lead to immediate loss of life and property, but also cause permanent physical disability and severe psychological trauma among survivors, resulting in reduction in quality of life [2,3].

In China, flood is one of the most common and severe forms of natural disasters. A catastrophic flood struck Bazhong city in Sichuan province, China in September 2011 [4]. The flood caused 31 deaths and 160 injuries, and estimated 1.5 million residents were affected. The estimated direct economic loss was worth 4.5 billion RMB (about US$ 658.4 million). Bazhong is a relatively isolated place in Sichuan, there have been no major movements in and out of the village after the flood.

Surveys on health-related quality of life (HRQoL) among survivors are widely used to evaluate the health consequences of disasters [5-7]. Previous studies have assessed the impacts of floods in terms of mortality, morbidity, and posttraumatic stress disorder [8-10], but only a few reported the HRQoL among flood survivors [3,11,12], and none were specifically targeting the elderly. People aged over 60 are a vulnerable population whose life quality is sensitive physical and mental conditions, life styles and environmental changes [13,14]. External traumatic events such as floods could have further detrimental impacts on their life quality. This study aims to provide a timely and thorough assessment of post-disaster HRQoL and the underlying associated factors among elderly in the flood-affected Bazhong, Southwest China.

## Methods

Our study method was derived from the 4th national health services survey (2008) and employed a similar methodology. The 4^th^ national health services survey is a nationwide standardized survey, which has been conducted by the Chinese Ministry of Health every 5 years. The latest survey was conducted from mid-June to early July in 2008 (prior to the Bazhong flood). A multi-stage stratified random cluster sampling method has been adopted in this survey and Sichuan province was selected. The 4^th^ national survey used a household interview method as the main information collection approach. Qualified investigators visited sample households and interviewed all household members. Similar approach was also employed in our current study but only seniors aged >60 were included. Further, questionnaire used in our study was derived based on Family Health Questionnaire as part of the 4^th^ national health services survey.

### Study participants

A multistage random sampling was employed to recruit participants from four counties under the jurisdiction of Bazhong in February, 2012. There are only four counties in Bazhong and out of these counties,the eight villages are randomly selected out of the total 188 villages. From each village, we contacted the village administration and random selected 150 elderly people (aged > 60y) based on their demographic records. A total of 1200 elderly people were recruited from eight selected villages. The participants must be over the age of 60, have no cognitive dissonance, consciousness disturbance, significant disorder of physical, mental functions or communication barriers. The participation of the study was voluntary. Since, seventeen refused to participate, the number of valid survey was 1183 (98.6%). All participants had been living in their local villages since the outbreak of the 2011 flood till the day of this survey. In the absence of pre-disaster relevant information in Bazhong, we used health status and HRQoL data of rural elderly in Sichuan province from the 4th national health services survey (2008) as a reference for comparison [15,16].

### Data collection

Data were collected through face-to-face interviews conducted by trained investigators from the Nantong University, who received training to identify cognitive difficulties. During the interview, interviewees who cannot communicate consciously were excluded and replaced with a new individual selected from the residence registration list. All personal information of participants was de-identified and recorded anonymously. Three categories of indicators, including socio-demographic characteristics, health status, and HRQoL were collected.

### Socio-demographic characteristics

Socio-demographic characteristic indicators included age, gender, occupation, level of education, marital status, economic status, living style, tobacco consumption (constant smokers, occasional smokers, and non-smokers), alcohol consumption (constant drinkers, occasional drinkers, and non-drinkers), sleep quality (poor, moderate and good), and family relationship (inharmonious, ordinary, harmonious) were collected.

### Health status

Measurements on health status included two-week healthcare-seeking rate and chronic disease prevalence. This part of the questionnaire was designed based on the validated 2008 National Health Services Survey in China [17]. The two-week healthcare-seeking rate is defined as the proportion of individuals who have sought for healthcare services in the past two week from the day of the survey. Chronic disease prevalence refers to the proportion of being diagnosed with chronic diseases (including hypertension, gastroenteritis, disc herniation, rheumatoid arthritis, diabetes mellitus and so on) within six-month prior to the survey. All health status indicators were self-reported.

### Health-related quality of life

HRQoL was measured using the SF-36 health questionnaire [18]. SF-36 consisted of eight different domains related to quality of life with a total of 36 items: (1) physical functioning (PF, 10 items); (2) role limitations due to physical illness (RP, 4 items); (3) bodily pain (BP, 2 items); (4) general health perceptions (GH, 5 items); (5) vitality (VT, 4 items); (6) social functioning (SF, 2 items); (7) role limitations due to emotional problems (RE, 3 items); and (8) mental health (MH, 5 items). The Physical Component Summary (PCS = PF + RP + BP + GH) and Mental Component Summary (MCS = VT + SF + RE + MH) were calculated. The score in each domain of the SF-36 was linearly transformed in to a standard score, ranging from 0 to100, with a higher score reflecting better self-perceived health [18]. SF-36 has been previously validated to be a reliable survey for elderly in China [19,20].

### Statistical analysis

Chi-square test was used to compare prevalence rates. HRQoL scores of study subjects and the reference group (rural Sichuan elderly [15,16]) were compared using nonparametric Mann–Whitney test. HRQoL score with standardized Z-statistics ≤ −1.0 was considered as “poor HRQoL” [21,22]; otherwise it is considered as “good HRQoL”. Univariate logistic regression analysis was conducted to assess the independent association between the HRQoL score and participants’ socio-demographic characteristics, factors with *p* < 0.1 were included in multivariate logistic regression. Dependent variable was poor HRQoL. Non-parametric Spearman correlation analysis was applied to examine the relationship between PCS and MCS scores. All data were analyzed using the Statistical Package for Social Sciences (SPSS) version 17.0.

### Ethical considerations

This study was reviewed and approved by the Human Research Ethics Committee of the Nantong University, Jiangsu Province, China. The objectives and the procedure of the study, and potential risks and benefits of participating in the study were given to potential participants during the recruitment of study subjects. Verbal and written consent procedures were given to the study participants and they had the right to discontinue the survey at any time.

## Results

The mean age of the 1183 participants was 68.9 ± 7.8 years (range 60–99) and male-to-female ratio was 1.4:1. Of these, 1005 were married (85.0%), but only 275 of whom (27.4%) were living with their spouses at the time of survey. Most of the participants were peasants (79.9%), and received primary education (73.8%) or below. About one-third (36.9%) of the participants regarded their economic status as poor, whereas 52.1% and 11.0% stated ‘moderate’ and ‘good’. Constant smokers and drinker accounted for 50.4% and 48.9% of the sample respectively. Only 36 participants (3.0%) reported inharmonious family relationship (Table 1).

**Table 1:** **Socio**-**demographic characteristics for subjects included in analysis**

**Demographic characteristics**	**Number** **(Percent, %)**
**Age** **(years)**	
60-79	1043 (88.2)
80-99	140 (11.8)
**Sex**	
Male	696 (58.8)
Female	487 (41.2)
**Vocation**	
Farmer	945 (79.9)
No farmer	238 (20.1)
**Years of Education**	
0-6	873 (73.8)
7-9	260 (22.0)
10 or over	50 (4.2)
**Marital Status**	
Singlehood	178 (15.1)
Married	1005 (85.0)
**Economic Status**	
Poor	437 (36.9)
Moderate	61 (52.1)
Good	130 (11.0)
**Tobacco Consumption**	
Constant Smokers	596 (50.4)
Occasional Smokers	298 (25.2)
Non-smokers	289 (24.4)
**Alcohol Consumption**	
Constant drinkers	579 (48.9)
Occasional drinkers	404 (34.2)
Nondrinkers	200 (16.9)
**Family Relationship**	
Inharmonious	36 (3.0)
Ordinary	331 (28.0)
Harmonious	816 (69.0)

### Health status

The two-week healthcare-seeking rate among post-flood Bazhong elderly (59.3%) was significantly higher (χ2 = 5.134, *p* = 0.013) in comparison with the rural Sichuan elderly (55.7%). Notably, acute upper respiratory tract infection accounts for 52.0% of the reported disease cases among Bazhong elderly. However, post-flood Bazhong elderly demonstrated a significantly lower prevalence of chronic disease than the reference group (33.2% versus 44.4%, χ2 = 48.847, *p* < 0.001).

### Health-related quality of life

**Figure 1:**
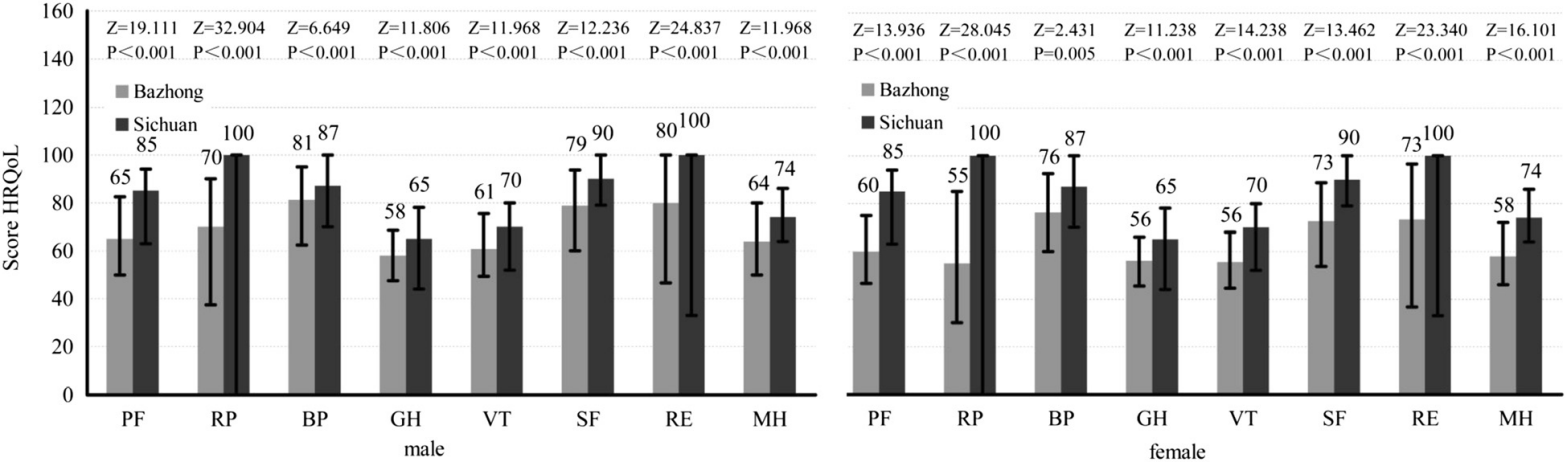
**Comparison of HRQoL scores** (**M**, **IQR**) **measured by SF**-**36 both male and female of elderly after flood disaster between Bazhong and Sichuan.** (PF-Physical Functioning; RP-Role Limitations Due to Physical Health Problems; GH-General Health Perceptions; BP-Bodily Pain; VT-Vitality; SF-Social Functioning; RE-Role Limitations Due to Emotional Problems; MH-Mental Health.

**Figure 2:**
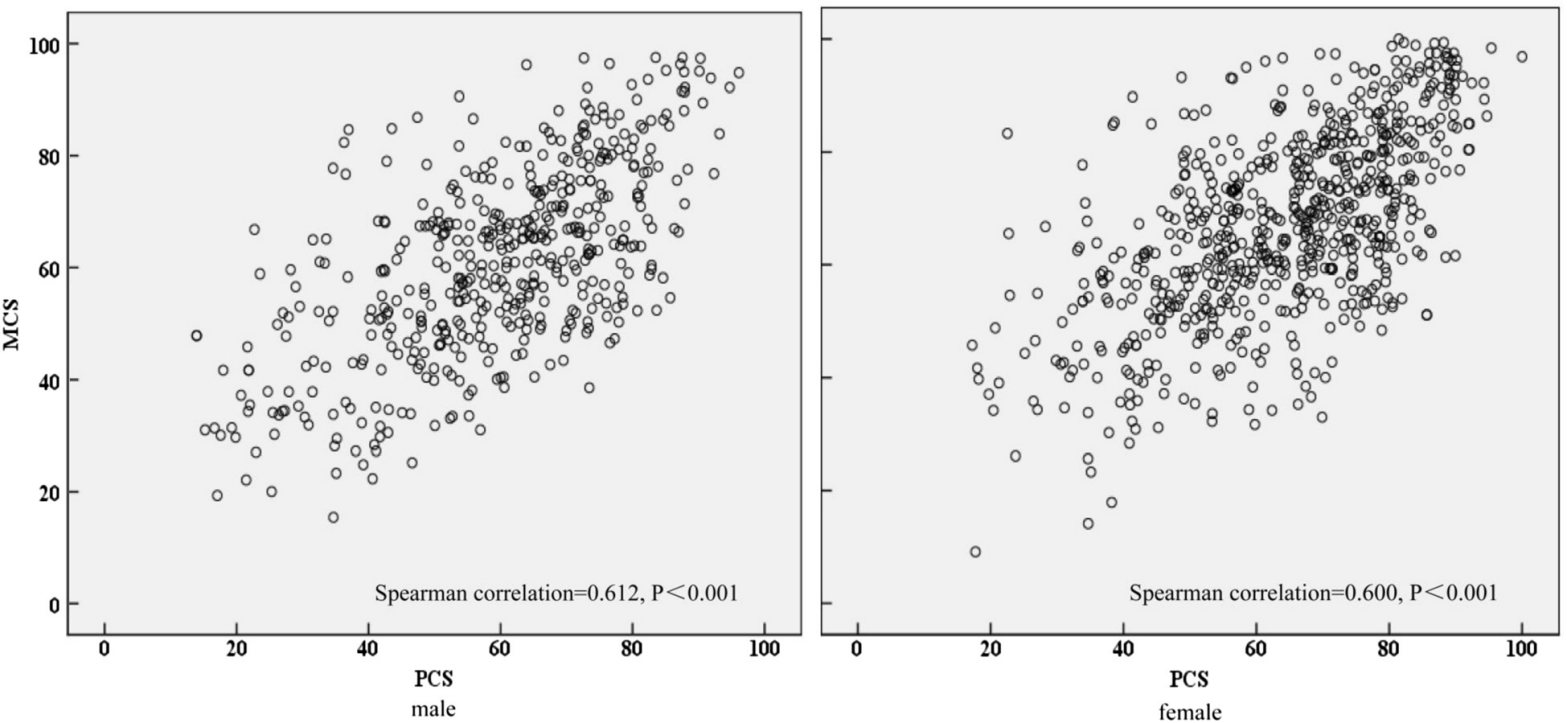
**Spearman correlation between Physical Component Summary** **(PCS)** **and Mental Component Summary** **(MCS)** **among post-** **flood elderly male and female.**

The overall median HRQoL score was 64.5 (IQR: 53.3-74.2), whereas the respective summary scores for the overall physical health (PCS) and mental health (MCS) were 64.2 (51.2-75.3) and 64.9 (53.1-77.1). The score of bodily pain was the highest (78.8 [61.3-93.8]), followed by role limitations due to emotional problems (76.7 [43.3-100.0]) and social functioning (76.3 [58.8-92.5]). The median score of physical functioning, role limitations due to physical health problems, mental health and vitality scores were 62.5 (48.5-79.0), 62.5 (35.0-87.5), 62.0 (48.0-78.0) and 59.0 (47.0-73.0), respectively. The general health perceptions has the lowest score (57.0 [46.5-67.5]). Notably, all dimension scores among Bazhong elderly were significantly lower than the rural elderly in Sichuan (Figure 1). Multivariate regression analysis showed that poor physical conditions was associated with older age, singlehood, poor sleep patterns, chronic diseases, being hospitalized in the past year and living alone (Table 2). In addition to these factors, being female and being sick in the past two weeks also significantly associated with poor mental health. Correlations between physical and mental health were significantly in both genders (*Spearman*, Male: *r* = 0.612, *p* < 0.001; female: *r* = 0.600, *p* < 0.001, respectively; Figure 2).

**Table 2:** **Logistic regression analysis of determinants of poor health**-**related quality of life in elderly after flood disaster**

	**Physical Component Summary** **(PCS)**					**Mental Component Summary** **(MCS)**				
	**Median** **(IQR)**	**Univariate analysis**		**Multivariate analysis**		**Median** **(IQR)**	**Univariate analysis**		**Multivariate analysis**	
		**OR**	**95%** **CI**	**OR**	**95%** **CI**		**OR**	**95%** **CI**	**OR**	**95%** **CI**
**Age (years)**										
60-79	65.7 (52.5-76.1)	1		1		65.9 (54.7-78.2)	1		1	
80-99	54.8 (42.2-68.9)	2.825^c^	1.909 ~ 4.181	2.331^c^	1.535 ~ 3.539	53.4 (42.8-65.6)	2.932^c^	1.96 ~ 4.368	2.536^c^	1.610 ~ 3.994
**Sex**										
Male	66.4 (52.7-77.4)	1				67.2 (56.5-79.3)	1		1	
Female	61.2 (48.6-72.9)	1.540^b^	1.135 ~ 2.090			61.0 (49.7-73.1)	1.906^c^	1.39 ~ 2.615	1.754^b^	1.232 ~ 2.499
**Vocation**										
Farmer	64.4 (51.2-75.1)	1				64.9 (53.4-77.4)	1			
No farmer	63.6 (51.0-76.2)	1.067	0.734 ~ 1.552			64.9 (52.1-76.2)	1.073	0.729 ~ 1.578		
**Years of Education**										
0-6	63.0 (49.8-74.5)	1				63.9 (52.5-75.9)	1			
7-9	67.8 (54.4-76.7)	0.681	0.457 ~ 1.015			67.8 (54.0-79.2)	0.944	0.644 ~ 1.383		
10 or over	69.2 (54.2-76.5)	0.862	0.397 ~ 1.872			69.2 (57.5-79.3)	0.451	0.160 ~ 1.274		
**Marital Status**										
Singlehood	52.8 (40.8-65.8)	1		1		51.2 (39.7-61.9)	1		1	
Married	66.2 (53.4-76.6)	0.308^c^	0.215 ~ 0.440	0.403 ^c^	0.276 ~ 0.590	66.9 (56.3-78.9)	0.174 ^c^	0.122 ~ 0.249	0.225^c^	0.152 ~ 0.333
**Economic Status**										
Poor	63.0 (49.5-74.8)	1				61.9 (51.1-75.4)	1			
Moderate	65.2 (52.5-76.6)	0.821	0.594 ~ 1.136			65.9 (54.4-78.9)	0.725	0.519 ~ 1.013		
Good	61.3 (51.0-71.5)	0.895	0.533 ~ 1.503			67.4 (55.1-75.9)	0.873	0.516 ~ 1.479		
**Tobacco Consumption**										
Constant Smokers	64.0 (50.5-74.6)	1				64.4 (51.3-75.8)	1			
Occasional Smokers	63.8 (50.1-75.7)	0.904	0.629 ~ 1.298			63.9 (53.5-76.9)	0.954	0.659 ~ 1.381		
Non-smokers	66.0 (54.0-76.6)	0.589^c^	0.391 ~ 0.886			67.6 (56.1-79.9)	0.568^c^	0.370 ~ 0.872		
**Alcohol Consumption**										
Constant drinkers	63.6 (49.8-74.5)	1				63.4 (52.3-74.6)	1			
Occasional drinkers	65.8 (52.5-76.8)	0.742	0.527 ~ 1.044			67.3 (53.9-80.1)	0.907	0.642 ~ 1.281		
Nondrinkers	63.6 (52.2-75.4)	0.651	0.413 ~ 1.026			65.0 (54.5-77.3)	0.669	0.415 ~ 1.080		
**Family Relationship**										
Inharmonious	56.4 (44.5-67.4)	1				64.6 (53.3-73.8)	1			
Ordinary	64.2 (51.5-73.7)	0.651	0.291 ~ 1.456			63.4 (51.1-74.8)	1.486	0.556 ~ 3.972		
Harmonious	64.5 (51.4-76.2)	0.579	0.266 ~ 1.259			65.2 (53.9-78.1)	1.027	0.392 ~ 2.696		
**Sleep patterns**										
Poor	56.5 (42.0-71.1)	1		1		56.9 (42.8-67.1)	1		1	
Moderate	65.7 (52.7-75.6)	0.383^c^	0.266 ~ 0.553	0.456^c^	0.310 ~ 0.670	66.8 (54.9-77.9)	0.322^c^	0.221 ~ 0.469	0.442 ^c^	0.291 ~ 0.672
Good	66.3 (53.6-77.9)	0.336^c^	0.224 ~ 0.506	0.393^c^	0.256 ~ 0.603	66.5 (55.5-79.7)	0.285^c^	0.187 ~ 0.434	0.368^c^	0.231 ~ 0.586
**Illnesses within Two Weeks**										
Yes	59.5 (45.7-73.0)	1				59.4 (43.1-70.8)	1		1	
No	65.8 (53.1-76.5)	0.523^c^	0.379 ~ 0.722			66.6 (55.1-78.6)	0.290^c^	0.209 ~ 0.401	0.392^c^	0.265 ~ 0.578
**Chronic Disease**										
Yes	57.9 (43.8-72.6)	1		1		55.9 (44.8-66.5)	1		1	
No	65.5 (52.7-76.5)	0.465^c^	0.333 ~ 0.649	0.606^b^	0.416 ~ 0.884	67.1 (56.0-78.8)	0.339^c^	0.243 ~ 0.474	0.512^a^	0.345 ~ 0.761
**One-year Hospitalized**										
Yes	57.6 (43.2-72.9)	1		1		54.7 (42.5-69.3)	1			
No	64.7 (52.4-75.8)	0.425^c^	0.289 ~ 0.626	0.597^a^	0.383 ~ 0.931	65.7 (54.5-78.1)	0.288^c^	0.197 ~ 0.421		
**Living Style**										
Living alone	59.1 (47.2-70.9)	1		1		59.4 (48.5-81.8)	1		1	
Living with descendant	63.4 (50.6-74.6)	0.913	0.590 ~ 1.412	0.992	0.624 ~ 1.579	64.1 (51.6-75.7)	0.714	0.466 ~ 1.095	0.702	0.431 ~ 1.141
Living with spouse	67.9 (56.2-79.3)	0.365^c^	0.209 ~ 0.639	0.475^a^	0.264 ~ 0.856	68.1 (59.4-83.2)	0.273^c^	0.154 ~ 0.483	0.319^c^	0.169 ~ 0.603

Abbreviations: *OR*, odds ratio; *CI*, confidence interval.

^a^ *P* < 0.05, ^b^ *P* < 0.01, ^c^ *P* < 0.001.

## Discussion

This is the first study that investigates post-flood health status and HRQoL for elderly in China. Our results indicated that health status and HRQoL among the post-flood Bazhong elderly were significantly poorer than that of the rural elderly in Sichuan province, suggesting significant negative impacts of flood among the studied participants. This result was consistent with findings among the broader general population in other Chinese city and Korea [5,11]. Older age, singlehood, poor sleep patterns, chronic diseases, being hospitalized in the past year, living alone, being female and being sick in the past two weeks were significantly associated with poor HRQoL.

Flood may have increased both acute disease morbidities and hospitalization rate, which are significantly contributed to individuals’ poor quality of life. Floods are known to increase incidence of endemic infectious diseases, such as malaria and diarrhea [23]. Surveys in China conducted among non-elderly population also showed that the rate of infectious disease morbidity also increased after the floods [24,25]. Consistent with this, the high rate of two-week healthcare-seeking in Bazhong elderly might attribute to polluted drinking water, deterioration of living conditions, and difficulties in accessing medical services caused by the flood [23,24]. The frequently reported acute upper respiratory syndrome might be also associated with the emergence of acute infectious diseases. Acute symptoms may mainly contribute to the post-flood healthcare-seeking behavior than other chronic diseases. However, chronic diseases may lead to lingering physical pains and generate substantial psychological pressure and emotional instability due to long-term and expensive medical treatments [26,27].

Our study reported lower HRQoL scores among elderly than the reference Sichuan province and other parts of China [28]. Flood has greater detrimental impacts on female and single elderly [29,30]. The higher HRQoL score among male elderly in this survey may attribute to a number of reasons. First, men usually have a better physical and physiological conditions than women; and hence they are more adaptive to deteriorating living environment [31]. Second, women are found to react more negatively to the same traumatic events compared with men. Yilmaz et.al., investigated 393 female and 328 male in Kocaeli after the 1999 Izmit earthquake in Turkey. The study indicated that female participants experienced a greater emotional stress and trauma than male participants [32]. In addition, a review paper showed that female were more likely to develop post-traumatic stress disorder (PTSD), especially chronic PTSD, than male [33]. Married couples are more resistant to the negative impacts of flood. The fact that the HRQoL score of the married subjects was significantly higher than that of the single elderly indicated that daily care and mutual emotional support between spouses are beneficial to both their physical and mental health [34].

Mental and physical conditions are significantly correlated in both genders among post-flood elderly. Compromised physical conditions caused by the flood-induced diseases and the restraints of activities, may lead to emotional instabilities and depression, damaging their mental health [35]. In turn, damaged mental conditions, such as PTSD, would substantially affects victims’ daily activities and social interactions, and consequently cause damage in their physical health [36]. Although the causality between mental and physical conditions cannot be confirmed in this cross-sectional survey, this result implies that due attention should be given to both aspects of physical and mental health when providing healthcare to elderly in flood-affected areas in China.

This study has a number of limitations. First, health status and HRQoL of the participants are not directly comparable before and after the disaster because of the absence of pre-flood information. As a result, relevant data in rural Sichuan elderly residents were adopted for comparison. We acknowledged that demographic characteristics, educational level and poverty rates of Bazhong elderly are comparable to those of rural Sichuan. However, their potential underlying effects on the general health of Bazhong elderly population were not investigated in this study. Second, this study was conducted in a selected flood-affected area in Bazhong city. Due to the differences in economic status and ability of disaster relief, its findings may not be generalized to other flood-affected areas in China. Third, the cross-sectional design of this study prevents us from drawing any causality conclusions.

Study findings suggest a marked decline in health status among elderly in Bazhong after the 2011 flood. The study has important implications to health policy and post-flood management for the elderly in a rural Chinese setting. Although prevention of infectious diseases outbreaks remains the main focus of post-flood management, measures that minimize other detrimental impacts of flood on elderly should also be included. In particular, interventions targeting elderly need to be sensitive to their age, gender and married status. Further resources should be specifically allocated to those diagnosed with chronic diseases and without family support.

## Conclusion

Our study suggested a likely decline in health status among elderly in Bazhong after the 2011 flood. Post-flood management targeting elderly need to be sensitive to their age, gender, married status and status of chronic diseases.

## Abbreviations

HRQoL: Health-related quality of life
MOS SF-36: Medical Outcomes Study Short Form-36
GDP: Gross Domestic Product
RP: Physical illness
BP: Bodily pain
GH: General health perceptions
VT: Vitality
SF: Social functioning
RE: Role limitations due to emotional problems
MH: Mental health
PCS: Physical Component Summary
MCS: Mental Component Summary
PTSD: Post-traumatic stress disorder
